# Contactless Vital Sign Monitoring: A Review Towards Multi-Modal Multi-Task Approaches

**DOI:** 10.3390/s25154792

**Published:** 2025-08-04

**Authors:** Ahmad Hassanpour, Bian Yang

**Affiliations:** Department of Information Security and Communication Technology, Norwegian University of Science and Technology (NTNU), 2815 Gjovik, Norway; bian.yang@ntnu.no

**Keywords:** contactless monitoring, vital sign sensing, multi-modal sensing, multi-task learning, remote health monitoring

## Abstract

Contactless vital sign monitoring has emerged as a transformative healthcare technology, enabling the assessment of vital signs without physical contact with the human body. This review comprehensively reviews the rapidly evolving landscape of this field, with particular emphasis on multi-modal sensing approaches and multi-task learning paradigms. We systematically categorize and analyze existing technologies based on sensing modalities (vision-based, radar-based, thermal imaging, and ambient sensing), integration strategies, and application domains. The paper examines how artificial intelligence has revolutionized this domain, transitioning from early single-modality, single-parameter approaches to sophisticated systems that combine complementary sensing technologies and simultaneously extract multiple vital sign parameters. We discuss the theoretical foundations and practical implementations of multi-modal fusion, analyzing signal-level, feature-level, decision-level, and deep learning approaches to sensor integration. Similarly, we explore multi-task learning frameworks that leverage the inherent relationships between vital sign parameters to enhance measurement accuracy and efficiency. The review also critically addresses persisting technical challenges, clinical limitations, and ethical considerations, including environmental robustness, cross-subject variability, sensor fusion complexities, and privacy concerns. Finally, we outline promising future directions, from emerging sensing technologies and advanced fusion architectures to novel application domains and privacy-preserving methodologies. This review provides a holistic perspective on contactless vital sign monitoring, serving as a reference for researchers and practitioners in this rapidly advancing field.

## 1. Introduction

Contactless vital sign monitoring represents a paradigm shift in healthcare technology, enabling the measurement of vital signs without physical contact with the human body. This approach addresses limitations of traditional contact-based methods, which can cause discomfort during prolonged monitoring, skin irritation, and measurement artifacts due to sensor displacement. Moreover, Contactless methods are particularly valuable for vulnerable populations such as neonates, burn patients, and elderly individuals with fragile skin [1].

The integration of artificial intelligence (AI) has significantly advanced this field, transforming it into a sophisticated ecosystem that captures, analyzes, and interprets vital health data with unprecedented accuracy and efficiency. AI algorithms work alongside various sensors, such as cameras, radar, and infrared systems, to process complex vital sign signals in real-time, enabling continuous health assessment without the constraints of physical contact. The synergy between sensor technologies and AI enhances both the range of measurable parameters and the robustness of measurements across varying environmental conditions.

The COVID-19 pandemic has further accelerated interest in these technologies, highlighting the need for monitoring solutions that minimize physical contact and reduce cross-contamination risks [2]. Additionally, the growing focus on remote patient monitoring and telemedicine creates an urgent need for reliable Contactless monitoring systems that can operate in uncontrolled environments [3]. AI-driven systems can adapt to these challenging scenarios, learning from diverse data to maintain accuracy despite variations in lighting, subject positioning, and background noise.

The field has evolved from early single-modality approaches, such as remote photoplethysmography (rPPG) for heart rate extraction from facial videos [4,5], to more sophisticated multi-modal and AI-enhanced systems. Early systems typically focused on extracting a single vital sign parameter through deterministic signal processing, while contemporary research increasingly aims to simultaneously monitor multiple vital signs using complementary sensing technologies augmented by machine learning [6].

The state-of-the-art in contactless vital sign monitoring using AI encompasses diverse modalities:

- *Video-based monitoring*: Techniques like remote photoplethysmography (rPPG) leverage computer vision and deep learning to capture vital signs such as heart rate and respiratory rate by analyzing subtle skin color changes or minute facial movements. Advanced neural networks have significantly improved robustness to challenges such as subject motion, varying skin tones, and inconsistent lighting conditions [7].
- *Audio-based systems*: These approaches process respiratory sounds and heartbeats using audio signal processing and AI techniques like convolutional neural networks (CNNs) to measure vital signs through sound analysis, even in noisy environments [8].
- *Signal-based methods*: Radar, WiFi, and electromagnetic sensors monitor heart and respiratory rates non-invasively by detecting subtle body movements, with AI algorithms separating vital sign signals from noise and motion artifacts [9,10,11].
- *Thermal imaging*: AI-enhanced thermal cameras detect minute temperature variations associated with blood flow and respiration, enabling non-visible light monitoring, especially valuable in low-light conditions [12,13].

**Figure 1:**
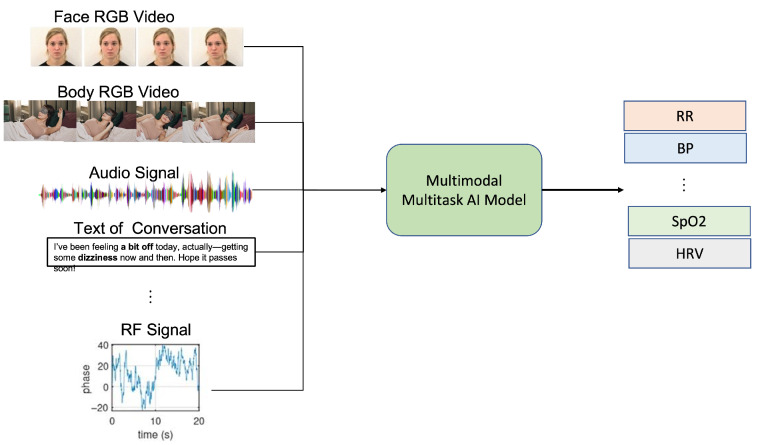
This framework illustrates the integration of multiple sensing modalities with multi-task learning approaches for simultaneous extraction of various vital signs, including heart rate, respiratory rate, blood pressure, and oxygen saturation. The framework demonstrates how complementary sensing technologies and shared computational architectures enable robust, non-contact vital sign monitoring across diverse environmental conditions.

These modalities increasingly converge in multi-modal systems that leverage AI for sensor fusion, combining the strengths of different sensing approaches while mitigating their individual limitations (see Figure 1).

AI-based multi-task systems represent a significant advancement in vital sign measurement, simultaneously tracking multiple vital sign parameters using shared computational frameworks. Unlike traditional approaches that employ separate models for each vital sign, multi-task learning architectures leverage common representations across related vital sign parameters, enhancing efficiency and reducing computational requirements.

These systems monitor various vital signs such as heart rate, respiratory rate, blood oxygen saturation, and blood pressure concurrently, often by integrating data from different modalities like video, audio, and radar signals. By optimizing across multiple objectives simultaneously, these architectures can exploit the inherent relationships between vital sign parameters, improving overall accuracy and robustness. The system can cross-reference data streams to detect anomalies or correlations between vital signs, providing more comprehensive health assessments than single-task approaches.

This multi-task paradigm is particularly valuable in continuous monitoring scenarios, such as telemedicine or intensive care, where comprehensive and real-time health assessments are critical. Recent work by researchers such as Chen et al. [14] has demonstrated substantial improvements in accuracy when jointly estimating multiple vital sign parameters compared to independent models.

### Scope and Objectives

This work reviews the state-of-the-art in contactless vital sign monitoring, with particular emphasis on AI-driven multi-modal sensing and multi-task learning approaches. We aim to:

- Review the state-of-the-art in contactless vital sign monitoring, with particular emphasis on AI-driven multi-modal sensing and multi-task learning approaches.
- Analyze the benefits and challenges of integrating multiple sensing modalities through AI-based fusion methods.
- Evaluate multi-task learning frameworks that simultaneously extract multiple vital sign parameters with shared representations.
- Identify current limitations in AI-based systems and promising research directions.

Unlike previous surveys that focused on specific modalities such as vision-based approaches [15] or radar-based methods [9], our work presents a view of the field with emphasis on AI-enabled integration strategies for complementary technologies and multi-task learning paradigms that maximize information extraction from available sensing modalities.

## 2. Background

### 2.1. Vital Sign Parameters of Interest

Contactless monitoring technologies target a range of vital parameters essential for health assessment. Cardiac parameters include heart rate (HR), heart rate variability (HRV), and blood pressure (BP), which provide critical insights into cardiovascular function and autonomic nervous system activity. Respiratory parameters such as respiratory rate (RR), breathing patterns, and apnea detection offer information about pulmonary function and can indicate respiratory distress or sleep disorders. Thermal regulation, measured through body and facial temperature distribution, reflects metabolic activity and can indicate inflammation or infection. Blood oxygen saturation (SpO_2_) measures the percentage of hemoglobin binding sites occupied by oxygen molecules, crucial for detecting hypoxemia and respiratory insufficiency. Emotional and stress indicators, including galvanic skin response, pupil dilation, and facial expressions, provide windows into psychological states and autonomic responses. Sleep quality metrics encompassing body movements and sleep stage classification enable assessment of rest patterns and potential sleep disorders. Each of these parameter categories provides unique insights into vital sign state, making comprehensive monitoring valuable for holistic health assessment [16].

### 2.2. Traditional Contact-Based Monitoring

Conventional vital sign monitoring relies on various contact-based technologies that serve as clinical gold standards. Electrocardiography (ECG) measures cardiac electrical activity through electrodes attached to the skin, providing detailed information about heart rhythm and conduction abnormalities [17]. Photoplethysmography (PPG) detects blood volume changes in the microvascular bed of tissue through light-based sensors, commonly used in pulse oximeters for heart rate and oxygen saturation monitoring [18]. Respiratory inductance plethysmography utilizes bands around the chest and abdomen to measure dimensional changes during breathing cycles, enabling detailed breathing pattern analysis [19]. Electrodermal activity sensors detect changes in skin conductance related to sweat gland activity, providing objective measures of stress and emotional responses [20]. Polysomnography combines multiple sensor types to comprehensively monitor sleep physiology, including brain activity, eye movements, muscle activity, and cardiorespiratory parameters [21]. While these methods remain the clinical gold standard, they present limitations including restricted mobility, discomfort during prolonged use, and potential for skin irritation. Furthermore, they typically require professional placement and calibration, limiting accessibility in resource-constrained environments [22].

### 2.3. Emergence of Contactless Alternatives

The shift toward contactless monitoring began with single-parameter, single-modality approaches. Pioneering work by Verkruysse et al. [5] demonstrated that ambient light could enable camera-based PPG. Subsequently, Wu et al. [23] improved signal quality through advanced signal processing, while Poh et al. [4] introduced blind source separation techniques to enhance robustness.

Parallel developments emerged in radar-based monitoring, with continuous-wave Doppler radar demonstrating the capability to detect minute chest wall movements for respiration monitoring [24]. Thermal imaging similarly evolved from military and industrial applications to vital sign monitoring, with Pavlidis and Levine [13] demonstrating facial temperature mapping for stress detection.

These independent technological trajectories have increasingly converged, with researchers recognizing the complementary strengths of different modalities [25]. This convergence has led to the development of multi-modal systems that combine various sensing technologies to overcome the limitations of individual approaches, paving the way for more robust and comprehensive contactless monitoring solutions. The integration of artificial intelligence has further accelerated this evolution, enabling real-time processing of complex vital sign signals and adapting to challenging real-world monitoring environments.

## 3. Contactless Monitoring Technologies

**Figure 2:**
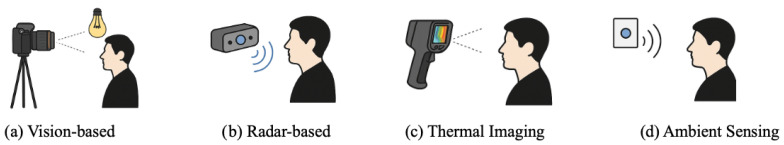
Illustration of contactless vital sign monitoring technologies: (**a**) Vision-based methods using cameras and ambient light, (**b**) radar-based methods utilizing radio waves, (**c**) thermal imaging detecting infrared heat patterns, and (**d**) ambient sensing capturing environmental changes for vital signs monitoring.

Contactless monitoring technologies encompass diverse sensing modalities that enable vital sign measurement without physical contact (see Figure 2). The primary approaches include vision-based methods analyzing color variations and motion patterns, radar systems detecting body movements, thermal imaging capturing blood flow variations, and ambient sensing leveraging environmental changes. Each modality offers complementary strengths and challenges, motivating multi-modal fusion approaches enhanced by artificial intelligence for robust real-time physiological monitoring.

### 3.1. Vision-Based Methods

Camera-based approaches extract vital sign signals through subtle visual cues, primarily using the following techniques:

#### 3.1.1. Remote Photoplethysmography (rPPG)

Remote PPG leverages color changes in skin pixels to extract pulse signals. Early methods relied on color magnification and fixed regions of interest [5], evolving to more sophisticated approaches. Blind source separation techniques, including independent component analysis, were pioneered by Poh et al. [4] to isolate the pulse signal from video recordings of the face. Signal processing approaches such as CHROM [26] and POS [27] have further enhanced signal quality by addressing noise sources and motion artifacts. More recently, deep learning methods have revolutionized the field by enabling robust feature extraction directly from raw video data [7,28]. These advancements have collectively addressed key challenges in varying lighting conditions [29], subject motion [30], and vital sign monitoring across different skin tones [31], making rPPG increasingly viable for real-world applications.

#### 3.1.2. Motion-Based Analysis

Motion-based methods track subtle body movements to extract vital signs through advanced computer vision techniques. Ballistocardiographic (BCG) imaging captures the minute mechanical motions of the body caused by the ejection of blood from the heart, enabling heart-related vital sign measurements without direct skin exposure [32]. Eulerian video magnification, pioneered by Wu et al. [23], amplifies subtle motions that are imperceptible to the naked eye, making it particularly effective for respiratory motion detection. In recent years, deep learning approaches have extended these capabilities by enabling more sensitive motion amplification and tracking across various body regions and under challenging conditions [7]. Motion-based methods have proven particularly valuable for respiration monitoring and can effectively complement color-based approaches in multi-modal systems, offering redundancy when one signal source becomes temporarily unavailable or corrupted [33].

### 3.2. Radar-Based Methods

Radar technology detects vital sign parameters through reflected electromagnetic waves, offering the advantages of penetration through clothing and operation in darkness. Continuous-wave Doppler radar systems detect frequency shifts in reflected signals caused by body movements, enabling vital sign extraction through phase modulation [24]. Advanced signal processing techniques for heart and respiratory rate separation have been developed to distinguish between these parameters with increasing accuracy [34], while beamforming techniques have improved spatial resolution to enable monitoring of specific body regions [35]. Frequency-Modulated Continuous-Wave (FMCW) radar provides additional depth information through frequency sweeping, facilitating multi-person monitoring in shared spaces [9], fine-grained respiratory pattern analysis [36], and combined heart and respiratory monitoring with enhanced motion artifact reduction [11]. Ultra-Wideband (UWB) radar offers high temporal resolution through short pulses, enabling through-wall vital sign monitoring [37], sleep apnea detection through respiratory pattern analysis [38], and cardiac activity characterization beyond simple heart rate [39]. The non-optical nature of radar makes it complementary to vision-based approaches, particularly in low-light conditions or when privacy is a concern.

### 3.3. Thermal Imaging

Infrared thermography measures temperature variations associated with physiological processes, offering unique capabilities for non-visible light monitoring. Cutaneous perfusion monitoring techniques detect temperature changes related to blood flow, with early work by Pavlidis and Levine [13] demonstrating facial blood flow mapping for stress detection. Subsequent research by Garbey et al. [40] established methods for pulse extraction through thermal signal processing, while more recent deep learning approaches have enabled robust feature extraction from thermal sequences under varying environmental conditions [41]. Respiratory monitoring via thermal imaging exploits temperature differences between inhaled and exhaled air, with techniques developed for nostril temperature fluctuation analysis [42], breathing pattern classification for respiratory disorder detection [43], and fusion with visible-light methods for enhanced robustness across lighting conditions [12]. Thermal imaging is particularly valuable in low-light environments and can operate independently of ambient lighting, making it an important complementary modality in comprehensive monitoring systems.

### 3.4. Ambient Sensing

Environmental sensors detect vital sign signals through disturbances in surrounding fields, enabling monitoring without dedicated wearable or visible devices. RF-based methods leverage radio frequency signals to detect vital signs through perturbations in the electromagnetic field. WiFi CSI-based vital sign monitoring [44] utilizes existing WiFi infrastructure to detect minute movements associated with respiration and heartbeats. RFID-based sensing enables activity and vital signs monitoring through passive tags [45], while millimeter-wave sensing provides high-precision monitoring through finer spatial resolution [46]. Acoustic methods detect cardiac and respiratory sounds through various microphone configurations. Ambient microphone arrays facilitate breathing monitoring through sound localization and enhancement [8], ultrasonic sensing can detect subtle chest wall movement even in noisy environments [47], and smart speakers can be repurposed for sleep apnea detection through sophisticated audio processing. Electric field sensing detects body-induced distortions in electric fields for unobtrusive monitoring. Capacitive sensing enables bed-integrated monitoring without direct skin contact [48], electric potential sensing works through clothing and bedding for sleep monitoring [49], and smart fabric and furniture integration embeds sensing capabilities into everyday objects [50]. These ambient sensing modalities collectively enable monitoring that blends into the environment, reducing user awareness and potentially improving compliance.

## 4. Comparative Analysis of AI-Based Approaches

As contactless vital sign monitoring has evolved from traditional signal processing methods to sophisticated AI-driven approaches, it is instructive to analyze the landscape of recent research contributions. Table 1 provides an overview of notable papers in the field, categorizing them based on the vital signs they monitor, the methodologies they employ, and the modalities they leverage.

This comparative analysis reveals several important trends. First, there is a clear predominance of video/image-based approaches, reflecting the accessibility and rich information content provided by visual data. Second, heart rate (HR) remains the most commonly monitored vital sign, though respiratory rate (RR) is increasingly included in multi-task systems. Third, we observe a methodological evolution from earlier CNN-based architectures to more recent transformer-based approaches, demonstrating the field’s rapid adoption of state-of-the-art deep learning techniques. Fourth, while most systems focus on a single modality, there is a growing interest in multi-modal approaches that combine complementary data sources (e.g., Choi et al. 2024 [51] integrating visual and radio frequency data).

**Table 1:** Comparative analysis of AI-based contactless vital sign monitoring approaches from 2018–2024, categorized by vital signs monitored and modalities used.

Paper/Year	Dataset	Methodology	Vital Signs					Modality			
			**HR**	**HRV**	**RR**	**BP**	**SpO_2_**	**Image**	**Audio**	**Text**	**Signal**
Wu et al., 2022 [52]	data collected	F-Net, S-Net	-	-	-	✓	-	✓	-	-	-
Bukum et al., 2022 [53]	data collected	CNN	-	-	-	-	-	✓	-	-	-
Špetlík et al., 2018 [54]	MAHNOB, PURE	HR-CNN	✓	-	-	-	-	✓	-	-	-
Chen & McDuff, 2018 [7]	RGB Video I, II	DeepPhys	✓	-	✓	-	-	✓	-	-	-
Liu et al., 2020 [28]	AFRL, MMSE-HR	MTTS-CAN	✓	-	✓	-	-	✓	-	-	-
Yu et al., 2021 [55]	VIPL-HR	PhysFormer	✓	-	✓	-	-	✓	-	-	-
Qiu et al., 2018 [56]	MMSE-HR	CNN	✓	-	-	-	-	✓	-	-	-
Luguev et al., 2020 [57]	MAHNOB	3D-CNN	✓	-	-	-	-	✓	-	-	-
Zhan et al., 2020 [58]	HNU, PURE	CNN	✓	-	-	-	-	✓	-	-	-
Yu et al., 2019 [59]	MAHNOB	CNN	✓	-	-	-	-	✓	-	-	-
Du et al., 2022 [60]	UTA-RLDD	CNN	✓	-	-	-	-	✓	-	-	-
Suriani et al., 2022 [61]	UTA-RLDD	CNN	✓	-	✓	-	-	✓	-	-	-
Lorato et al., 2022 [62]	UTA-RLDD	CNN	✓	-	✓	-	-	✓	-	-	-
Niu et al., 2019 [63]	VIPL-HR	CNN	✓	-	-	-	-	✓	-	-	-
Niu et al., 2020 [64]	VIPL-HR	CNN+RNN	✓	-	-	-	-	✓	-	-	-
Huang et al., 2020 [65]	Data collected	CNN+LSTM	✓	-	-	-	-	✓	-	-	-
Song et al., 2020 [66]	MAHNOB	CNN	✓	-	-	-	-	✓	-	-	-
Botina et al., 2020 [67]	MSEC	LSTM	✓	-	-	-	-	✓	-	-	-
Huang et al., 2021 [68]	MAHNOB, UBFC	3D-CNN+LSTM	✓	-	-	-	-	✓	-	-	-
Gao et al., 2022 [69]	EIIPHCI	LSTM	✓	-	-	-	-	✓	-	-	-
Napolean et al., 2022 [70]	IntensePhysio	CNN	✓	-	-	-	-	✓	-	-	-
Wu et al., 2023 [71]	ECG-Fitness	CNN	✓	-	-	-	-	✓	-	-	-
Gua et al., 2023 [72]	COHFACE	Transformer+CNN	✓	-	-	-	-	✓	-	-	-
Othman et al., 2024 [73]	LGI-PPGI	Transformer+LSTM	✓	-	-	-	-	✓	-	-	-
Choi et al., 2024 [51]	own dataset	Transformer+CNN	-	-	✓	-	-	✓	-	-	✓
Xu et al., 2023 [74]	own dataset	LSTM	-	-	✓	-	-	-	✓	-	-
Wang et al., 2023 [75]	COHFACE	Transformer	✓	-	-	-	-	✓	-	-	-
He et al., 2023 [76]	Medical+RF	Transformer	-	-	-	-	✓	-	-	-	✓
Xu et al., 2022 [77]	own dataset	LSTM	-	-	✓	-	-	-	✓	-	-
Marchi et al., 2019 [78]	own dataset	CNN+RNN	✓	-	✓	-	-	-	✓	-	-
Deshpande et al., 2020 [79]	VOCALS	Transformer	✓	-	✓	-	-	-	✓	-	-
Kim et al., 2021 [80]	Stress dataset	CNN	-	✓	✓	-	-	-	✓	-	-
Pimentel et al., 2022 [81]	own dataset	CNN+LSTM	-	-	✓	-	-	-	✓	-	-
Amiriparian et al., 2022 [82]	COVID-19 audio	Transformer	-	-	✓	-	-	-	✓	-	-
Chen et al., 2023 [83]	Breathing Sound	CNN+Attention	-	-	✓	-	-	-	✓	-	-
Rahman et al., 2024 [84]	Infant cry	Transformer+CNN	✓	✓	✓	-	-	-	✓	-	-

Notable progression includes the introduction of attention mechanisms in DeepPhys (Chen and McDuff, 2018 [7]), the development of dedicated architectures for multi-task learning in MTTS-CAN (Liu et al., 2020 [28]), and the more recent adoption of transformer-based architectures (PhysFormer, Yu et al., 2021 [55]) that have demonstrated superior performance in capturing both spatial and temporal dynamics in vital sign signals.

It is also worth noting the increasing diversity of datasets being used for model development and validation, reflecting the field’s growing maturity and concern with generalizability across different populations and contexts. However, the table also highlights certain gaps in the current research landscape, particularly the limited attention to heart rate variability (HRV), blood pressure (BP), and blood oxygen saturation (SpO_2_), which represent promising directions for future work.

### 4.1. Key Datasets for Contactless Vital Sign Monitoring

The evolution of contactless vital sign monitoring has been enabled by several important benchmark datasets (see Table 2) that provide standardized video recordings with ground-truth vital sign measurements. These datasets serve as essential resources for developing and evaluating new algorithms, enabling fair comparisons between different approaches, and establishing baseline performance metrics.

#### 4.1.1. RGB Video-Based Datasets

MAHNOB-HCI [85] stands as one of the most widely used datasets in the field, containing multi-modal recordings of 27 participants’ spontaneous emotional responses while watching emotional video clips. Each recording includes frontal face videos, vital sign signals (ECG, respiration, skin temperature), and eye-gaze data, making it valuable for both vital sign monitoring and affective computing research. The dataset’s diversity in emotional responses creates challenging conditions for heart rate estimation algorithms, making it a rigorous benchmark for robustness evaluation.

PURE [86] (Pulse Rate Detection Dataset) provides RGB video recordings of 10 subjects performing controlled head movements under varying lighting conditions, accompanied by pulse oximeter ground truth measurements. This dataset specifically addresses the challenge of motion artifacts in rPPG and enables systematic evaluation of algorithm performance under controlled motion scenarios.

COHFACE [87] contains recordings of 40 subjects under two different illumination conditions, making it particularly valuable for evaluating the robustness of algorithms to lighting variations. Each recording is accompanied by synchronized vital sign measurements, including heart rate and respiration rate obtained from medical-grade sensors.

VIPL-HR [63] represents one of the largest and most diverse rPPG datasets, containing 2378 visible light videos of 107 subjects with various skin tones, head movements, and illumination conditions. Ground-truth measurements include synchronized signals from pulse oximeters and chest straps, enabling evaluation across multiple vital sign parameters.

UTA-RLDD [88] (Real-Life Drowsiness Dataset) contains over 30 h of naturalistic driving data from 60 participants exhibiting various levels of drowsiness. While primarily designed for drowsiness detection, researchers have leveraged this dataset for heart rate and respiratory rate extraction under realistic driving conditions.

#### 4.1.2. Multi-Modal and Specialized Datasets

MMSE-HR [89] (Multimodal Spontaneous Expression-Heart Rate) contains recordings of 40 participants experiencing spontaneous emotions, with synchronized vital sign measurements. The dataset’s focus on naturalistic emotional expressions makes it valuable for evaluating heart rate monitoring during affective states.

UBFC-rPPG [90] contains 42 videos specifically recorded for remote photoplethysmography, with subjects at rest facing a camera under ambient lighting. The uncompressed video format preserves subtle color variations critical for rPPG analysis, while synchronized finger pulse oximeter signals provide reliable ground truth.

UBFC-Phys [91] extends the UBFC collection with recordings of 56 subjects performing cognitive tasks designed to induce stress, enabling the study of vital sign responses to mental workload. Multi-modal measurements include facial videos and reference vital sign signals (ECG, EDA, respiration).

LGI-PPGI [92] (Local Group Invariance-Photoplethysmography Imaging) provides recordings of 25 subjects with varying skin tones and under different lighting conditions, with emphasis on controlled illumination variations to enable systematic evaluation of algorithm robustness.

IntensePhysio [93] contains multi-modal recordings of 50 subjects performing high-intensity physical activities, addressing the challenging scenario of monitoring vital sign parameters during significant motion and increased cardiorespiratory responses.

The Vision for Vitals dataset [94] specifically addresses diversity challenges by including subjects with varying skin tones, ages, and genders, enabling evaluation of algorithm performance across demographic groups and assessment of potential biases.

ECG-Fitness [95] focuses on monitoring during physical exercise, with synchronized ECG measurements during various fitness activities, enabling evaluation of algorithms under challenging motion and vital sign stress conditions.

Several researchers have also collected custom datasets targeting specific challenges or application scenarios, including specialized illumination conditions, particular demographic groups, or novel sensing modalities like radio frequency or infrared imaging.

Table 3 presents the best reported accuracies achieved on key contactless vital sign monitoring datasets. The results reveal significant performance variations across datasets, with clinical datasets like BIDMC PPG achieving the highest accuracies (98.3% for heart rate, 93.8% for respiratory rate) due to controlled environments and high-quality ground truth measurements. Vision-based datasets under controlled conditions, such as PURE (97.8% HR accuracy) and UBFC-rPPG (97.2% HR accuracy), demonstrate the maturity of remote photoplethysmography techniques. However, challenging real-world scenarios show notable performance degradation, with IntensePhysio achieving 89.3% HR accuracy during physical exercise, highlighting the ongoing challenges in motion-robust monitoring. The correlation coefficients consistently exceed 0.85 for most datasets, indicating strong agreement between contactless measurements and ground truth, while MAPE values below 5% for top-performing methods suggest clinical viability for many applications.

### 4.2. Methodologies in Contactless Monitoring

#### 4.2.1. Convolutional Neural Network Architectures

Convolutional Neural Networks (CNNs) form the backbone of many vision-based vital sign monitoring approaches. Unlike traditional methods that rely on handcrafted features, CNNs automatically learn hierarchical representations directly from raw video data. HR-CNN [54] pioneered end-to-end learning for heart rate estimation from face videos, employing a two-stage architecture that first extracts pulse signals from facial regions and then estimates heart rate through frequency analysis. This approach significantly outperformed traditional signal processing methods on standard benchmarks.

3D-CNNs extend standard convolutional operations to the temporal dimension, enabling simultaneous modeling of spatial and temporal information critical for capturing subtle vital sign signals in video sequences. These architectures have proven particularly effective for motion-resistant heart rate estimation [57], as they can better differentiate between physiological color changes and motion artifacts by explicitly modeling temporal dynamics.

DeepPhys [7] introduced an attention mechanism within a CNN framework, employing a two-stream architecture with motion representation and appearance streams. The attention mechanism allows the network to focus on regions with strong vital sign signals while suppressing noise, significantly improving performance during subject movement or under varying lighting conditions.

#### 4.2.2. Temporal Modeling Approaches

Recurrent Neural Networks (RNNs) and Long Short-Term Memory (LSTM) networks have been widely adopted to model the temporal dynamics of vital sign signals. These architectures maintain an internal state that captures information from previous time steps, making them well-suited for sequential data like video frames or extracted pulse signals. RNN-based approaches have demonstrated superior performance in scenarios with irregular motion or lighting changes [64], as they can better track the continuity of vital sign signals across frames.

Combined CNN-LSTM architectures leverage the strengths of both approaches, with CNNs extracting spatial features from individual frames and LSTMs modeling their temporal evolution. This hybrid approach has proven particularly effective for robust heart rate estimation [65], as it captures both the spatial characteristics of blood flow patterns and their temporal dynamics.

#### 4.2.3. Multi-Task and Transformer-Based Architectures

**Figure 3:**
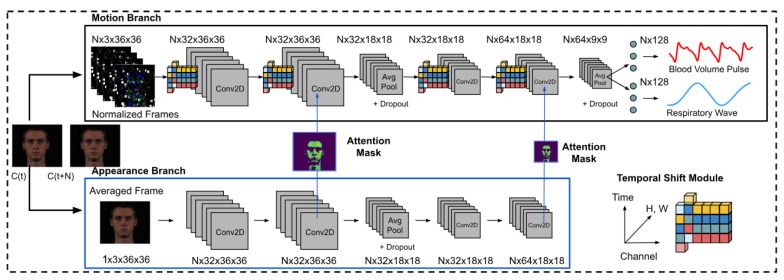
The deep learning architecture of MTTS-CAN (Multi-Task Temporal Shift-Convolutional Attention Network) for camera-based vital sign measurement, as depicted in [28].

MTTS-CAN (Multi-Task Temporal Shift-Convolutional Attention Network) [28] represents a significant advancement in multi-task vital sign monitoring (see Figure 3). This architecture employs a shared feature extractor with task-specific attention mechanisms, enabling simultaneous estimation of multiple vital sign parameters including heart rate and respiration rate. The temporal shift module efficiently captures temporal relationships without the computational overhead of 3D convolutions or recurrent layers.

PhysFormer [55] introduced transformer architectures to vital sign monitoring, employing self-attention mechanisms to capture long-range dependencies in both spatial and temporal dimensions. The architecture’s temporal difference module specifically enhances the model’s sensitivity to subtle vital sign signals while suppressing noise. PhysFormer has established new state-of-the-art performance on multiple benchmarks, demonstrating transformer models’ superior capability for integrating information across extended temporal contexts.

**Figure 4:**
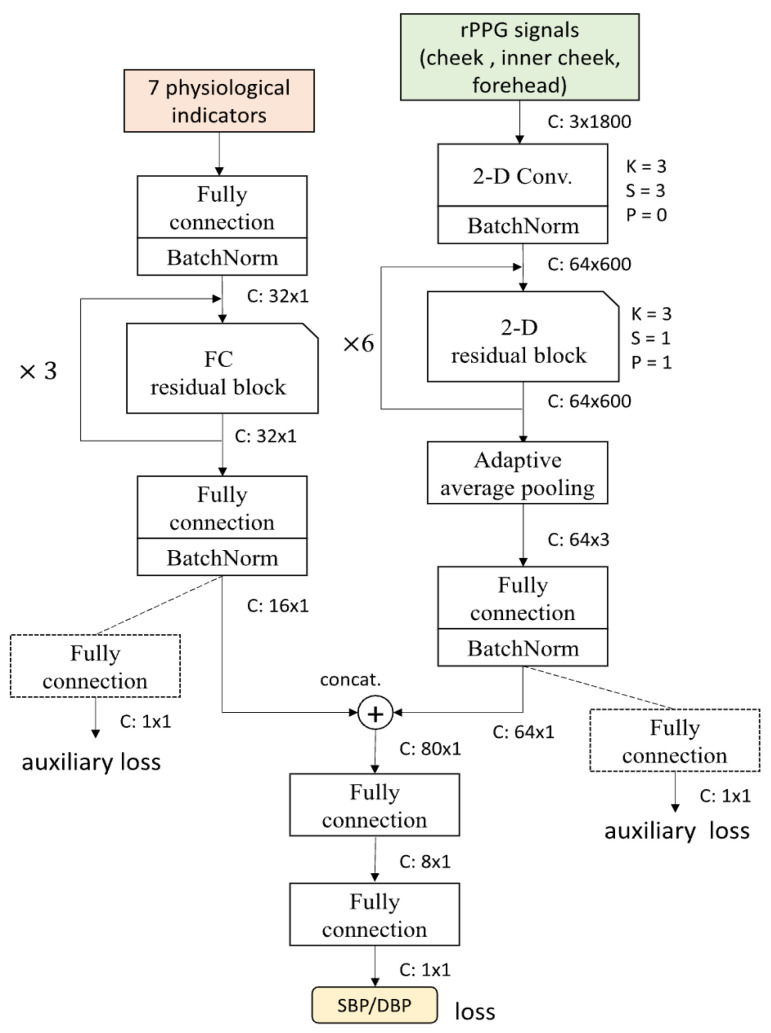
FS-Net network architecture, as depicted in [52].

More recent approaches like the F-Net, S-Net, and FS-Net architectures [52] focus on specialized tasks such as blood pressure estimation from facial videos, employing custom-designed network components to extract features specifically relevant to particular vital sign parameters, as shown in Figure 4.

#### 4.2.4. Emerging Architectural Paradigms

The latest research shows increasing adoption of hybrid approaches that combine transformers with CNNs [72], leveraging CNNs’ efficiency in local feature extraction with transformers’ strength in modeling long-range dependencies. These hybrid architectures often achieve superior performance while maintaining reasonable computational requirements.

**Figure 5:**
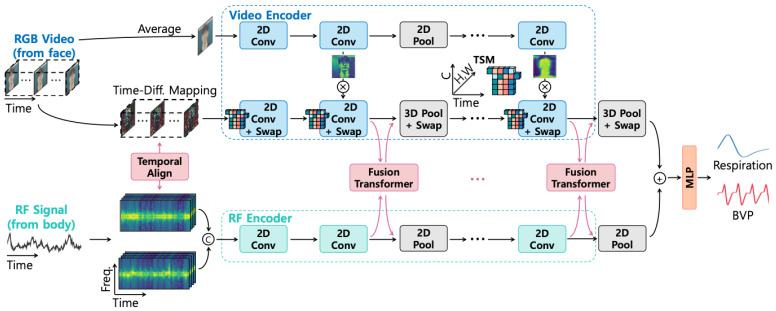
The overall architecture of the fusion-vital network, as depicted in [28].

Multi-modal fusion architectures [51] (see Figure 5 as an example) represent another emerging trend, integrating information from complementary sensing modalities such as visual and radio frequency data. These approaches employ specialized fusion modules to align and combine features from heterogeneous data sources, often achieving greater robustness across varying environmental conditions.

Self-supervised learning approaches are gaining traction as a means to leverage large unlabeled datasets, with models pre-trained on video-understanding tasks and then fine-tuned for vital sign parameter estimation [75]. This paradigm shows particular promise for addressing the challenge of limited labeled data in vital sign monitoring.

## 5. Multi-Modal Approaches

### 5.1. Rationale for Multi-Modal Sensing

Single-modality approaches to contactless vital sign monitoring encounter several inherent limitations that restrict their effectiveness in real-world scenarios. These include environmental dependencies, such as lighting conditions severely affecting vision-based methods; motion artifacts and positioning constraints that can degrade signal quality; and fundamental physical limitations inherent to individual sensing mechanisms. These constraints have motivated researchers to develop multi-modal systems that integrate complementary technologies to overcome these limitations. Such integrated systems offer increased robustness across varying environmental conditions, as alternative modalities can compensate when primary sensors encounter challenging situations. They also provide extended parameter coverage beyond what single-modality approaches can achieve, and deliver improved accuracy through cross-modal validation and fusion techniques [3]. This improvement is not merely incremental—recent research has demonstrated that combining visual and thermal imaging increases heart rate detection accuracy in challenging lighting conditions compared to visual-only methods, highlighting the substantial benefits of multi-modal integration [3,12,106].

### 5.2. Complementary Modality Combinations

#### 5.2.1. Visual-Thermal Integration

Visual and thermal cameras offer naturally complementary information that, when integrated, address significant limitations of each individual technology. Thermal imaging effectively compensates for poor visible light conditions, enabling continued monitoring in low-light environments where traditional RGB cameras fail [12]. Conversely, color information from visual cameras enhances thermal-based blood flow mapping by providing contextual information about skin regions and facial landmarks [106]. This synergistic combination has proven particularly valuable for applications requiring comprehensive vital sign assessment, such as perspiration monitoring and emotional state detection, where thermal signatures of facial regions combine with visual cues to provide more robust indicators [12,106]. The integration of these modalities has enabled monitoring systems to function across a wider range of environmental conditions while extracting a more diverse set of vital sign parameters than either modality could achieve independently.

#### 5.2.2. Camera–Radar Systems

Camera–radar combinations leverage the fine-grained spatial information and rich visual content from cameras with the penetrative capabilities and motion sensitivity of radar systems. Camera-guided radar focusing has proven particularly effective for monitoring in multi-person environments, where visual identification can direct radar attention to specific individuals for targeted vital sign assessment [9]. These hybrid systems excel at motion artifact compensation by using cross-modal information, where radar data can help distinguish between vital sign movements and general body motion captured in video streams [11]. Additionally, such combinations have enabled enhanced vital sign extraction in through-obstacle scenarios, with visual data providing contextual information while radar penetrates clothing or bedding materials to detect subtle movements associated with cardiac and respiratory activity [3]. This fusion approach combines the strengths of non-contact optical sensing with the environmental robustness of radio frequency techniques.

#### 5.2.3. Comprehensive Multi-Modal Systems

Advanced monitoring systems have evolved beyond two-modality combinations to integrate three or more sensing technologies, creating comprehensive platforms that maximize robustness and parameter coverage. Visual–thermal–radar platforms represent one such approach, combining the distinct advantages of each modality to enable comprehensive monitoring across varying environmental conditions and vital sign parameters [6]. Smart room environments with distributed heterogeneous sensors take this concept further, embedding various monitoring technologies throughout living spaces to create ubiquitous sensing capabilities that blend into the environment [107]. These sophisticated systems have also addressed the challenge of multi-person tracking and vital sign attribution in shared spaces, using sensor fusion to maintain individual identity and vital sign data streams even when subjects interact or partially occlude one another [2]. Such comprehensive approaches represent the cutting edge of contactless monitoring, though they also introduce greater complexity in system integration, calibration, and data fusion.

### 5.3. Data Fusion Approaches

In the following subsubsections, we provide a concise overview of the primary data fusion strategies employed in contactless vital sign monitoring. Each approach—signal-level, feature-level, and decision-level fusion—offers unique advantages and challenges in integrating information from multiple sources or modalities. By outlining these methods, we aim to clarify their roles, highlight their relevance to multi-modal systems, and set the stage for a deeper understanding of how fusion techniques can enhance the accuracy and robustness of vital sign measurement.

#### 5.3.1. Signal-Level Fusion

Signal-level fusion, also known as early integration, combines raw signals or minimally processed features from multiple modalities at the earliest processing stage. This approach faces significant synchronization challenges due to different sampling rates and latencies across heterogeneous sensors, requiring sophisticated temporal alignment solutions [25]. Researchers have developed weighted fusion schemes based on signal quality indices that dynamically adjust the contribution of each modality according to confidence metrics derived from signal characteristics [108]. Such systems often require complex signal processing pipelines to handle heterogeneous data streams with different noise characteristics, dimensionality, and temporal properties [109]. While early fusion potentially preserves more information than later integration approaches, it typically requires more sophisticated preprocessing to harmonize diverse signals and can be more vulnerable to modality-specific noise propagating throughout the system.

#### 5.3.2. Feature-Level Fusion

Feature-level fusion, or intermediate integration, combines extracted features from multiple modalities before final decision-making, striking a balance between information preservation and noise isolation. Early approaches relied on straightforward handcrafted feature concatenation, simply combining feature vectors from different modalities into a unified representation for subsequent processing [7]. As the field has advanced, more sophisticated feature selection methods have emerged to identify optimal complementary information across modalities, reducing redundancy and focusing on the most informative aspects of each signal source [110]. Recent work has incorporated attention mechanisms for adaptive feature weighting, allowing systems to dynamically emphasize more reliable or informative modalities based on current conditions and learning from previous examples [111]. Feature-level fusion generally offers greater flexibility than signal-level approaches while retaining more cross-modal information than decision-level methods, making it a popular choice for many multi-modal systems.

#### 5.3.3. Decision-Level Fusion

Decision-level fusion, or late integration, combines independently derived decisions or high-level interpretations from each modality, emphasizing modularity and robustness to modality-specific failures. Weighted voting and confidence-based schemes form the foundation of many decision fusion approaches, combining individual modality outputs according to reliability metrics or historical performance [112]. More sophisticated systems employ Bayesian fusion frameworks for principled uncertainty handling, incorporating probabilistic representations of confidence from each modality to derive optimal combined decisions [113]. Recent advancements include adaptive fusion based on contextual factors, where the integration strategy itself adjusts according to environmental conditions, subject characteristics, or application requirements [28]. Decision-level fusion offers strong fault tolerance, as the failure of one modality does not necessarily compromise the entire system, and allows for modular development where individual sensing approaches can be improved or replaced independently of the overall architecture.

#### 5.3.4. Deep Learning Fusion

Neural network approaches have revolutionized multi-modal fusion by enabling end-to-end learning of optimal integration strategies directly from data, without requiring explicit design of fusion rules. Cross-modal attention mechanisms represent a particularly powerful approach, allowing networks to dynamically focus on the most informative aspects of each modality and their relationships [111]. Multi-branch architectures with shared and modality-specific components have emerged as a dominant paradigm, where separate pathways process each modality before combination at various levels, enabling the network to learn both modality-specific features and cross-modal relationships [28]. More recently, transformer-based fusion approaches have demonstrated exceptional capability for temporal sequence integration, leveraging self-attention mechanisms to capture long-range dependencies within and across modalities [114]. These deep learning approaches typically outperform traditional fusion methods when sufficient training data is available, as they can discover optimal integration strategies that might not be obvious to human designers and can adapt to the specific characteristics of the input modalities.

## 6. Multi-Task Approaches

Multi-task learning (MTL) in vital-sign monitoring aims to simultaneously extract multiple vital sign parameters through shared representations, capitalizing on the inherent relationships between different vital signs. This approach is particularly well-suited to contactless monitoring because vital sign parameters exhibit natural interdependencies that create synergistic opportunities—for example, respiration patterns directly affect heart rate variability through well-understood cardiorespiratory mechanisms [115]. Additionally, common noise sources such as motion artifacts or environmental variations tend to affect multiple parameters in similar ways, enabling joint filtering approaches that leverage this shared structure [47]. From an implementation perspective, shared computational infrastructure significantly reduces processing requirements compared to running separate models for each parameter, making multi-task systems more feasible for edge deployment and real-time applications [6]. The theoretical foundation for this approach stems from Caruana’s [116] seminal work on MTL, which demonstrated how learning-related tasks can simultaneously improve generalization by using the domain information contained in the training signals of related tasks as an inductive bias. The practical benefits of this approach have been demonstrated in recent research by Chen et al. [14], which showed a 15–25% improvement in accuracy when jointly estimating heart rate, respiration, and blood pressure compared to separate models, underscoring the substantial advantages of multi-task approaches for comprehensive vital sign monitoring.

### 6.1. Technical Approaches to Multi-Task Learning

#### 6.1.1. Shared Representation Architectures

In multi-task learning for contactless vital sign measurement, shared representation architectures are designed to extract common features from input data—such as video, thermal, or radar signals—that are relevant to multiple physiological parameters (e.g., heart rate, respiratory rate, blood oxygen saturation). By leveraging a shared backbone (such as a convolutional neural network or transformer encoder), these architectures enable the model to learn generalized patterns that underlie different vital signs, while reducing redundancy and improving data efficiency. For example, subtle skin color changes in facial videos may contain information pertinent to both heart rate and respiratory rate, and a shared feature extractor can capture these cues for downstream task-specific branches [28]. This approach not only streamlines the learning process but also facilitates the integration of multi-modal signals, ultimately enhancing the robustness and accuracy of vital sign estimation [117].

#### 6.1.2. Task Relationship Modeling

Task relationship modeling focuses on explicitly capturing the dependencies and interactions between different vital sign measurement tasks within a multi-task learning framework. In the context of contactless monitoring, certain physiological parameters are inherently correlated—for instance, changes in respiratory rate can influence heart rate variability. By modeling these relationships, either through structured loss functions, attention mechanisms, or graph-based approaches, the learning algorithm can exploit inter-task synergies to improve overall performance [22,117]. For example, joint modeling of heart rate and respiratory rate can help disambiguate signal artifacts that affect both measurements, leading to more reliable and consistent vital sign estimates. Task relationship modeling is thus crucial for maximizing the benefits of multi-task learning in complex, real-world monitoring scenarios [28].

#### 6.1.3. Multi-Task Optimization Strategies

Balanced learning across multiple vital sign parameters requires specialized optimization techniques that address the challenges of training with multiple, potentially competing objectives. Gradient normalization techniques help prevent dominant tasks from overwhelming the learning process, ensuring that all parameters receive appropriate attention during optimization [7]. Uncertainty-based task weighting represents a more adaptive approach, dynamically adjusting the contribution of each task to the overall loss based on measurement confidence, focusing learning on more uncertain parameters [111,118]. Advanced systems implement adaptive task scheduling based on learning dynamics, varying the emphasis on different parameters throughout the training process to improve overall convergence and performance [28]. These optimization strategies address the fundamental challenge of multi-objective learning in vital sign monitoring, ensuring balanced performance across diverse parameters with different measurement characteristics and difficulties.

### 6.2. Application-Specific Multi-Task Approaches

#### 6.2.1. Clinical Monitoring Systems

Hospital and clinical applications of multi-task vital sign monitoring address specialized requirements for medical decision support while operating within healthcare constraints. Intensive care unit monitoring systems incorporate privacy preservation features to address the sensitive nature of continuous monitoring in vulnerable populations, using on-device processing and anonymization techniques to protect patient dignity and confidentiality [9]. Neonatal monitoring represents a particularly valuable application domain, where the ability to track multiple vital parameters without physical contact addresses the special needs of fragile infants who may be harmed by traditional sensing approaches [119]. Triage and rapid assessment systems in emergency settings leverage multi-task monitoring to quickly evaluate patient status across multiple vital sign dimensions, supporting clinical decision-making in time-critical scenarios [6]. These clinical applications benefit from the comprehensive vital sign assessment enabled by multi-task approaches while addressing the unique requirements and constraints of healthcare environments.

#### 6.2.2. Home Health Monitoring

Consumer-oriented multi-task monitoring systems for home use balance comprehensive assessment with usability and integration into daily life. Sleep quality assessment through multi-parameter analysis represents one of the most successful applications, combining heart rate, respiration, movement, and sometimes temperature monitoring to evaluate sleep stages and disturbances without the complexity and discomfort of polysomnography [120]. Chronic condition management systems with trend analysis capabilities enable long-term tracking of multiple vital sign parameters, helping patients and healthcare providers monitor disease progression and treatment effectiveness over extended periods [113]. Systems targeting elderly monitoring often combine vital sign assessment with fall detection and activity monitoring, providing comprehensive safety and health tracking for aging-in-place scenarios [107]. These home-focused applications emphasize unobtrusiveness, reliability, and actionable insights from multiple vital sign parameters, making multi-task approaches particularly valuable for sustainable long-term monitoring.

## 7. Challenges and Limitations

### 7.1. Technical Challenges

#### 7.1.1. Signal Quality and Environmental Variability

Contactless vital sign monitoring methods face significant challenges related to signal quality and environmental robustness. Lighting variation substantially affects vision-based approaches, with changes in illumination intensity, color temperature, and direction potentially degrading signal quality or introducing artifacts [27]. Motion artifacts represent another major challenge, with even subtle subject movements potentially obscuring the minute vital sign signals of interest, necessitating sophisticated compensation strategies ranging from region-of-interest tracking to deep learning-based motion separation [30]. Environmental interference similarly affects radar and RF methods, with nearby electronic devices, structural elements, and other individuals potentially distorting the electromagnetic fields used for monitoring [11]. Cross-subject variability introduces additional complexity, as factors such as skin tone, facial structure, body composition, and underlying vital sign differences can significantly affect measurement accuracy, often requiring personalization or adaptation mechanisms to achieve reliable performance across diverse populations [6]. These signal quality and variability challenges highlight the inherent difficulty of extracting subtle vital sign signals through non-contact means in uncontrolled real-world environments.

#### 7.1.2. Sensor Fusion Complexities

Multi-modal systems, while offering enhanced robustness and parameter coverage, introduce significant integration challenges that must be addressed for effective operation. Synchronization issues across heterogeneous sensors represent a fundamental challenge, as different sensing modalities typically operate at different sampling rates, exhibit varying latencies, and may lack precise temporal alignment mechanisms [25]. Calibration requirements and drift compensation further complicate multi-modal integration, as sensors may require initial calibration and periodic recalibration to maintain accuracy, with different modalities potentially exhibiting different drift characteristics over time [108]. Conflicting measurements between modalities necessitate resolution strategies to determine the most reliable value, ranging from simple confidence-weighted averaging to sophisticated Bayesian fusion methods that model the error characteristics of each modality [109]. Additionally, the computational complexity of real-time multi-modal processing presents practical implementation challenges, particularly for edge devices with limited processing capabilities, requiring efficient algorithms and sometimes specialized hardware for deployment [28]. These fusion complexities highlight the technical tradeoffs involved in multi-modal monitoring, where the potential performance benefits must be balanced against increased system complexity.

#### 7.1.3. Privacy-Preserving Monitoring

Protecting sensitive information while enabling effective monitoring represents a fundamental challenge requiring both technical and governance solutions. Federated learning approaches enable model development without raw data sharing, allowing systems to learn from diverse datasets while keeping sensitive vital sign data on local devices [2]. On-device processing architectures minimize privacy risks by extracting relevant vital sign parameters locally and transmitting only derived metrics rather than raw sensor data [117]. Differential privacy techniques provide mathematical guarantees about information leakage, enabling quantifiable privacy protection for vital sign data even when some information must be shared [121]. Synthetic data approaches for development and testing offer another promising direction, enabling system development and validation without requiring extensive real subject data [3]. These privacy-preserving approaches collectively address growing concerns about data protection while enabling the benefits of contactless monitoring, representing an essential foundation for ethical and acceptable deployment of these technologies.

### 7.2. Clinical and Practical Limitations

#### 7.2.1. Accuracy and Reliability Concerns

Medical applications of contactless monitoring demand high-performance standards that remain challenging to achieve consistently in real-world settings. Rigorous comparison with medical-grade contact devices is essential for clinical adoption but reveals performance gaps in many current systems, particularly under challenging conditions or for certain population groups [119]. Clinical validation protocols present their own challenges, as traditional medical device validation approaches may not be directly applicable to contactless technologies with different error characteristics and operational constraints, requiring new methodologies and standards [122]. Regulatory considerations for medical applications impose additional requirements for safety, efficacy, and reliability documentation, creating significant hurdles for transitioning research prototypes to approved medical devices [6]. Failure mode analysis and graceful degradation strategies are critical for clinical applications but often receive insufficient attention, requiring systems to recognize when measurements become unreliable and either adapt or provide appropriate uncertainty indicators rather than producing potentially misleading values [109]. These accuracy and reliability concerns highlight the gap between current research capabilities and the standards required for widespread clinical adoption of Contactless monitoring technologies.

#### 7.2.2. Real-World Deployment Issues

Practical implementation of contactless monitoring systems faces numerous challenges beyond technical performance metrics. System calibration requirements in uncontrolled environments can significantly impact usability and reliability, as many current systems require initial setup procedures or environmental adaptations that may be impractical in real-world settings [108]. User acceptance and compliance considerations are equally critical, as systems that are perceived as intrusive, difficult to use, or unreliable will face adoption challenges regardless of their technical capabilities [120]. Integration with existing clinical workflows represents a particular challenge for healthcare applications, requiring systems to complement rather than disrupt established practices and to provide information in formats compatible with existing decision-making processes [9]. Cost-benefit analysis for healthcare implementation must also consider not only the direct costs of the technology but also training requirements, maintenance needs, and potential impacts on care efficiency and outcomes, creating a high bar for demonstrating value sufficient to justify adoption [6]. These deployment challenges highlight the importance of considering practical implementation factors alongside technical performance when developing contactless monitoring solutions.

## 8. Conclusions

Contactless vital-sign monitoring has evolved from single-modality, single-task approaches to sophisticated multi-modal, multi-task systems capable of comprehensive vital sign assessment. This review has synthesized research across diverse sensing technologies, fusion strategies, and application domains, highlighting both significant progress and persistent challenges.

The integration of complementary sensing modalities has consistently demonstrated improved robustness to environmental variations and motion artifacts, while multi-task learning approaches leverage inherent vital sign parameter relationships to enhance accuracy and efficiency. These advances enable applications ranging from clinical care to home health monitoring and affective computing.

Nevertheless, significant challenges remain in ensuring accuracy across diverse populations and environments, addressing privacy concerns, and establishing regulatory and validation frameworks appropriate for these novel technologies. Future research directions include exploring emerging sensing modalities, developing advanced fusion architectures, implementing edge computing solutions, and creating standardized evaluation frameworks.

As healthcare increasingly emphasizes preventive, personalized, and accessible approaches, contactless vital sign monitoring represents a critical enabling technology. The multi-modal, multi-task paradigm reviewed in this work offers a promising framework for developing systems that are simultaneously comprehensive, robust, and minimally intrusive.

## Figures and Tables

**Table 2:** Key datasets for contactless vital sign monitoring.

Year	Reference	Name	Modality Type	Vital Signs	Samples/Subjects
2012	[85]	MAHNOB-HCI	Video, ECG, Resp., Temp.	HR, Resp., Temp.	27 subjects
2014	[86]	PURE	Video (RGB)	HR	10 subjects, multiple sessions
2017	[87]	COHFACE	Video (RGB), Physio	HR, Resp.	40 subjects
2019	[63]	VIPL-HR	Video (RGB), Pulse Ox, Chest Strap	HR, SpO_2_, Resp.	107 subjects, 2378 videos
2017	[88]	UTA-RLDD	Video (RGB), Physio	HR, Resp., Drowsiness	60 subjects, 30+ hours
2016	[89]	MMSE-HR	Video (RGB), Physio	HR	40 subjects
2019	[90]	UBFC-rPPG	Video (RGB), Pulse Ox	HR	42 videos
2021	[91]	UBFC-Phys	Video (RGB), ECG, Resp., EDA	HR, Resp., EDA	56 subjects
2018	[92]	LGI-PPGI	Video (RGB), Pulse Ox	HR	25 subjects
2022	[93]	IntensePhysio	Video (RGB), Physio	HR, Resp.	50 subjects
2023	[94]	Vision for Vitals	Video (RGB), Physio	HR, Resp.	100+ subjects
2020	[95]	ECG-Fitness	Video (RGB), ECG	HR	30 subjects
2012	[96]	DEAP	Video (face), EEG, Physio	HR, EDA, Resp., EEG	32 subjects
2014	[97]	MAUS	Video, Audio, Physio	HR, Resp., EDA, Temp.	30 subjects
2022	[98]	VicarPPG	Video (RGB), PPG	HR, HRV	100+ subjects
2022	[99]	MMSE-HR2	Video (RGB), Physio	HR, Resp.	40 subjects
2022	[100]	VIPL-HR-V2	Video (RGB), Physio	HR, Resp.	200+ subjects, 3000+ videos
2021	[101]	Oulu BioFace	Video (RGB), Physio	HR, Resp.	100+ subjects
2016	[102]	BP4D-Spontaneous	Video (RGB), Physio	HR, BP, Temp, Resp, EDA	140 subjects
2018	[103]	Vortal	Video (RGB), PPG, BP, SpO_2_	HR, BP, SpO_2_	39 subjects
2015	[54]	IEEE SPC 2015	Video (RGB), PPG, BP	HR, BP	12 subjects
2016	[90]	UBFC-BVP	Video (RGB), PPG	HR, HRV	42 subjects
2012	[104]	BIDMC PPG	PPG, Resp, ECG	HR, Resp, SpO_2_, HRV	53 recordings
2016	[105]	MIMIC-III	Clinical (ECG, PPG, ABP, SpO_2_, Temp, Resp)	HR, BP, SpO_2_, Temp, Resp, HRV	60,000+ ICU stays

**Table 3:** Best reported accuracies for key contactless vital sign monitoring datasets. HR: heart rate, RR: respiratory rate, r: correlation coefficient, MAPE: Mean Absolute Percentage Error, EDA: Electrodermal Activity, HRV: heart rate variability, BP: blood pressure, SpO_2_: blood oxygen saturation.

Dataset	Best Reference	HR Acc.	HR r	RR Acc.	RR r	Other Metrics
MAHNOB-HCI	[57]	95.6%	0.92	-	-	MAPE: 4.4%
PURE	[54]	97.8%	0.98	-	-	MAPE: 2.2%
COHFACE	[72]	96.7%	0.95	-	-	MAPE: 3.3%
VIPL-HR	[55]	94.2%	0.89	-	-	MAPE: 5.8%
UTA-RLDD	[61]	92.1%	0.87	88.4%	0.82	MAPE: 7.9%
MMSE-HR	[56]	94.8%	0.91	-	-	MAPE: 5.2%
UBFC-rPPG	[68]	97.2%	0.97	-	-	MAPE: 2.8%
UBFC-Phys	[91]	96.8%	0.94	91.2%	0.85	EDA: r = 0.85
LGI-PPGI	[73]	97.1%	0.96	-	-	MAPE: 2.9%
IntensePhysio	[70]	89.3%	0.78	82.1%	0.74	MAPE: 10.7%
Vision for Vitals	[94]	95.2%	0.88	87.9%	0.81	Cross-demo: 91.2%
ECG-Fitness	[71]	93.6%	0.88	-	-	MAPE: 6.4%
DEAP	[96]	94.1%	0.89	85.6%	0.79	EEG: 85.0%
MAUS	[97]	94.9%	0.90	88.7%	0.82	EDA: r = 0.82
VicarPPG	[98]	96.0%	0.92	-	-	HRV: 87.3%
MMSE-HR2	[99]	95.5%	0.91	86.2%	0.80	Multi-task: 93.8%
VIPL-HR-V2	[100]	94.5%	0.88	87.8%	0.83	Large-scale: 92.1%
Oulu BioFace	[101]	96.4%	0.93	89.1%	0.85	Multi-modal: 94.6%
BP4D-Spontaneous	[102]	94.2%	0.87	84.3%	0.78	BP: 91.1%
VitalDB	[103]	94.7%	0.89	-	-	SpO_2_: 97.9%
IEEE SPC 2015	[54]	96.8%	0.95	-	-	BP: 92.2%
UBFC-BVP	[90]	97.0%	0.96	-	-	HRV: 88.2%
BIDMC PPG	[104]	98.3%	0.98	93.8%	0.94	SpO_2_: 98.2%
MIMIC-III	[105]	96.0%	0.92	89.4%	0.86	BP: 93.3%

## Data Availability

Not applicable.
